# *CPR* Gene Contributes to Integument Function and Ovary Development in a Rice Planthopper

**DOI:** 10.3390/ijms23052875

**Published:** 2022-03-06

**Authors:** Zhe-Chao Wang, Shuai Tao, Xu Cheng, Dan-Ting Li, Chuan-Xi Zhang, Yan-Yuan Bao

**Affiliations:** 1 Institute of Insect Sciences, Ministry of Agriculture Key Lab of Molecular Biology of Crop Pathogens and Insect Pests, College of Agriculture and Biotechnology, Zhejiang University, Hangzhou 310058, China; zhechaowang@zju.edu.cn (Z.-C.W.); shauishuai@zju.edu.cn (S.T.); 22016104@zju.edu.cn (X.C.); 2 Key Laboratory of Biology of Crop Pathogens and Insects of Zhejiang Province, Zhejiang University, Hangzhou 310058, China; 3 Zhejiang Provincial Key Laboratory of Biometrology and Inspection and Quarantine, College of Life Science, China Jiliang University, Hangzhou 310018, China; lidanting@cjlu.edu.cn; 4 State Key Laboratory for Managing Biotic and Chemical Threats to the Quality and Safety of Agro-products, Key Laboratory of Biotechnology in Plant Protection of Ministry of Agriculture and Zhejiang Province, Institute of Plant Virology, Ningbo University, Ningbo 315211, China

**Keywords:** *Nilaparvata lugens*, CPR, cuticular hydrocarbons, waterproof, water retention, ovary development

## Abstract

Nicotinamide adenine dinucleotide phosphate (NADPH)-cytochrome P450 reductase (CPR) is an essential enzyme that transfers electrons from NADPH to cytochrome P450 monooxygenases. CPR is involved in cuticular hydrocarbon (CHC) synthesis in insects and is vital for insect development and survival. Here, we clarify the physiological function of a *CPR* gene in *Nilaparvata lugens*, an important rice pest, by using RNA interference. *CPR* gene knockdown leads to the functional loss of waterproofing and water retention in the integument of female adults, which causes significantly reduced body weight and a lethal phenotype. Scanning electron microscopy shows that the lipid layer on the outermost surface of the abdominal cuticle becomes thin in ds*CPR*-injected adults. Furthermore, CHC profile analysis reveals that *CPR* knockdown significantly decreases the contents of CHCs with a carbon chain length ≥ C27 in adult females. Moreover, we find that *CPR* knockdown generates a deficient phenotype in ovaries with deformed oocytes and a complete failure of egg-laying. These findings suggest that *CPR* plays multiple functional roles in CHC biosynthesis and embryo development in insects.

## 1. Introduction

Nicotinamide adenine dinucleotide phosphate (NADPH)-cytochrome P450 reductase (CPR) is a cofactor of cytochrome P450 monooxygenases (P450s) that donates electrons from NADPH to the heme center of P450s [1,2]. CPR belongs to the flavoprotein family and contains an N-terminal transmembrane region and three conserved binding domains for flavin mononucleotide (FMN), flavin adenine dinucleotide (FAD), and NADPH [3,4,5]. CPRs are present in all living organisms. Several CPRs have been identified and characterized in insects, such as *Drosophila melanogaster* [6,7], *Helicoverpa armigera* [8], *Nilaparvata lugens* [9], *Rhopalosiphum padi* (L.) [10], *Laodelphax striatellus* (fallén) [11], *Locusta migratoria* [12], *Cnaphalocrocis medinalis* [12], *Spodoptera Litura* [13], *Bemisia tabaci* [14], *Acyrthosiphon pisum* [15], and *Diaphorina citri* [16]. 

P450 enzymes catalyze the oxidative metabolism of various endogenous and xenobiotic substrates, including insecticides, steroids, and cuticular hydrocarbons (CHCs). As cofactors of P450s, CPRs are also essential for the oxidative metabolism of different substrates. It is known that CPRs are closely related to susceptibility to insecticides and CHC biosynthesis in insects [7,17]. 

Insect CHCs are composed of straight and methyl-branched saturated alkanes and unsaturated alkenes. The lipid layer covering insect cuticles is composed of CHCs, which provide resistance to chemical compounds and prevent the movement of water through insect cuticles [17,18,19]. CHCs are generated from fatty acids, which are catalyzed by fatty acid synthases (FAS), elongases (ELO), desaturases, fatty acyl-CoA reductases (FAR), and CYP4G oxidative decarbonylase [20,21]. CYP4Gs belong to a subfamily of P450 enzymes, which are required for transferring the electron from CPR in the last step of CHC synthesis [7]. CHCs participate in the formation of the lipid layer, which provides the function of waterproofing and water retention in insects [22,23]. CPRs have also been found to be expressed in *Drosophila melanogaster* antennae and embryos at high levels [6], indicating that CPR may play an important role in embryo development. 

The brown planthopper, *Nilaparvata lugens* Stål (Hemiptera: Delphacidae), is one of the most destructive rice pests and is able to adapt to variable environments during long-distance migration [24,25]. By living in different humidities, such as extremely high humidities in paddy fields and volatile environments during migration, waterproofing and water retention are critical to *N. lugens* survival. Previous studies have reported that the knockdown of the *CPR* gene increases the susceptibility of *N. lugens* to insecticides [9]. However, it is unknown whether the *CPR* is involved in CHC synthesis during the development process in *N. lugens*.

In this study, we focus on the *CPR* gene to explore its functional roles in *N. lugens*. The expression profile of the *CPR* gene shows high transcript levels in the ovaries and eggs. Functional analysis using RNAi, waterproofing, and water retention testing, plus scanning electron microscopy combined with CHC profile analysis, revealed that CPR is involved in CHC biosynthesis and embryo development in *N. lugens*. Our findings are the first to report that CPR has important functions in oocyte development in female ovaries. Our data provide useful target molecules for the biological control of rice pests, which could be used to reduce pesticide usage in rice planting areas.

## 2. Results

### 2.1. Development Stage and Tissue Expression Specificities of the CPR Gene

**Figure 1:**
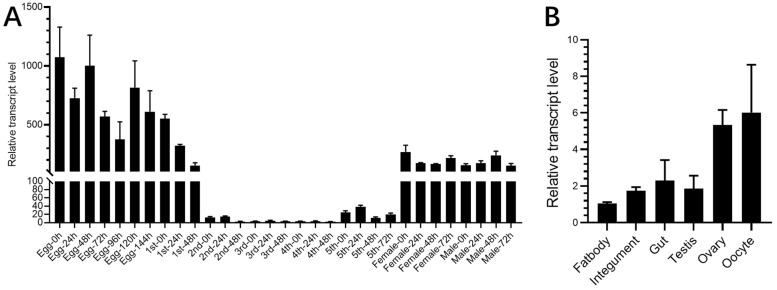
Spatial–temporal expression patterns of the *CPR* gene. (**A**) Relative transcript levels of *CPR* throughout the developmental stages of *N. lugens*. The 1st, 2nd, 3rd, 4th, and 5th refer to the 1st–5th instar nymphs. Female and male refer to adults. Egg refers to the laid eggs in the rice seedlings. n = 100 laid eggs, with 20 nymphs and 10 adults. (**B**) Relative transcript levels of *CPR* in different tissues. The fat bodies, guts, integuments, testis, ovaries, and oocytes were isolated from *N. lugens* adults. n = 50–100. The relative transcript levels of *CPR* were normalized using *N. lugens*18S rRNA threshold cycle (Ct) values. Mean ± SE were calculated from three biological replicates.

To explore the functional roles of the *CPR* gene, we first analyzed the developmental-stage expression using qRT-PCR. Throughout all developmental stages, the *CPR* transcripts were detected at very high levels in eggs laid from 0 to 144 h (Figure 1A). Moreover, high transcript levels were found in the 1st-instar nymphs at 0–48 h. Intriguingly, extremely low levels were detected in the 2nd–4th instar nymphal stages, and these gradually increased in the 5th-instar nymphal stage at 0–72 h. High transcript levels were then achieved after eclosion in the adults. The high transcript levels in the eggs and adults implicated that *CPR* might play a role during egg development and reproduction. Subsequently, we investigated the tissue-specific expression of *CPR* in adults. The highest transcript levels were detected in the ovaries and oocytes, while relatively low levels were detected in the fat bodies, integuments, guts, and testes. These data suggest that *CPR* may play an important role during the oocyte development in female ovaries (Figure 1B). Along with the development expression patterns found, we hypothesize that *CPR* is involved in oocyte maturation in the ovaries and egg development after oviposition. 

### 2.2. RNAi Result

**Figure 2:**
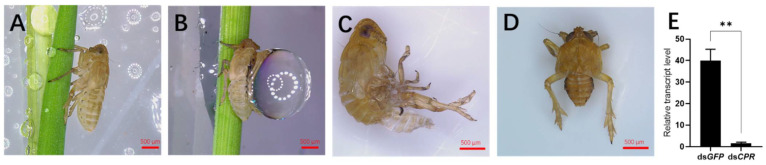
Phenotypes of RNAi. Each dsRNA was injected in the 4th-instar nymphs at 48 h after hatching. Phenotypes were observed in ds*GFP*- (**A**) and ds*CPR*-injected nymphs (**B**–**D**). (**E**) RNAi efficiency of the *CPR* gene, the asterisk ** indicate significant differences at *p* < 0.01 (Student’s *t*-test), difference from ds*GFP*. Mean ± SE were calculated from three biological replicates.

To understand the functions of the *CPR* gene in waterproofing and water retention, we performed RNAi by injecting ds*CPR* in the 4th-instar nymphs to knock down the gene expression. Waterproofing assay showed that no water droplets remained on the cuticles of the ds*GFP*-treated nymphs after spaying water on the insects (Figure 2A), suggesting their good waterproofing function; meanwhile, the cuticles of the ds*CPR*-injected nymphs were covered with water, forming a big water droplet (Figure 2B) and indicating that the waterproofing function was lost. A similar phenotype was observed when we soaked the dsRNA-treated insects in water. The ds*CPR*-injected nymphs that had been submerged were not able to move and surface from the water (Video S1); meanwhile, the ds*GFP*-treated nymphs kept afloat on the water and could jump to the wall of the glass tube (Video S2). We observed that the ds*CPR*-injected nymphs had molting deficiency (Figure 2C). These insects could not complete molting as the exuviae were stuck to the abdomen and legs. Moreover, some individuals could complete molting but showed an abnormal phenotype with a shrunk abdomen, which led to a lethal phenotype (Figure 2D). In contrast, the ds*GFP*-injected nymphs successfully completed molting. Those insects showed normal abdomens (Figure 2A). The qRT-PCR result confirmed the high interference efficiency of the *CPR* gene (Figure 2E).

**Figure 3:**
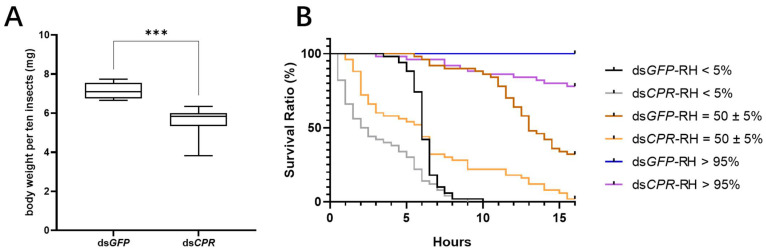
Observation of water retention in 5th-instar nymphs injected with dsRNA. (**A**) Measurement of body weights in dsRNA-treated adult females at 48 h after emergence under a relative humidity of 50 ± 5%. Body weights per ten insects were measured as a biological replicate. ds*GFP*-injected nymphs were used as a negative control. The data were calculated from nine biological replicates (mean ± SD). The asterisk *** indicate significant differences at *p* < 0.001 (Student’s *t*-test), difference from ds*GFP*. (**B**) Analysis of survival rates under different humidity treatments (relative humidity < 5%, =50 ± 5%, and > 95%) following dsRNA injection. Effect of different humidity treatments on the survival time of adult females of ds*CPR* or ds*GFP* in the 5th-instar nymphs at 48 h post injection. Fifty insect individuals were counted each 30 min in both treatments. Each humidity treatment showed *p* < 0.01 (Log-rank (Mantel–Cox) test), difference from ds*GFP*.

To understand the functions of the *CRP* gene in water retention, we measured the body weights of the dsRNA-injected insects at a relative humidity of 50 ± 5% and performed desiccation experiments by keeping the adult females under different humidity conditions at relative humidities of <5%, =50 ± 5%, and >95%. The body weights of the adult females that developed from ds*CPR*-injected 5th instar nymphs were significantly decreased when compared to those of the ds*GFP*-injected controls (Figure 3A). Under desiccation conditions at a relative humidity < 5%, the median survival time of ds*CPR*-treated insects, which was 2 h, was reduced by 2.5 times compared to that of the ds*GFP*-treated insects (6 h). Under the medium-humidity condition at a relative humidity of 50 ± 5%, the median survival time of the ds*CPR*-treated insects (6 h) was reduced by 2.17 times compared to that of the ds*GFP*-treated insects (13 h). Under the high-humidity condition at a relative humidity > 95%, 100% of the ds*GFP*-treated insects stayed alive for at least 16 h, while the ds*CPR*-treated insects started to die at 3 h and had a 20% mortality rate at 16 h (Figure 3B).

**Figure 4:**
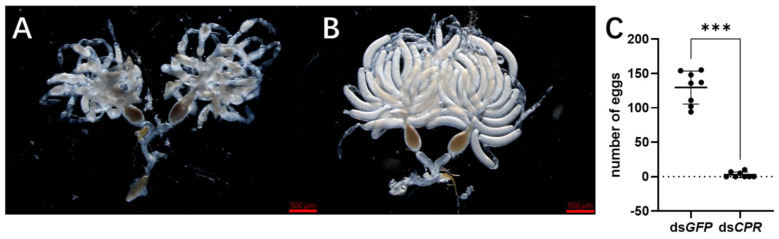
Phenotypes of RNAi. Each dsRNA was injected in the 5th-instar nymphs at 48 h after hatching. Ovaries of female adults were observed in ds*CPR*- (**A**) and ds*GFP*-injected insects (**B**). (**C**) Oviposition analysis. A dsRNA-injected female was mated with a non-injected male in each replicate and oviposition for 10 days. The eggs in the rice leaf sheaths were dissected and counted. Biological replicates were carried out for each mating (n = 8 ♀ × ♂). The statistical significance of the number of the laid eggs between the different treated groups was tested by a Student’s *t*-test. The asterisk *** indicate significant differences at *p* < 0.001.

Subsequently, we observed the ovaries of dsRNA-injected female adults. The ovaries of ds*CPR*-treated females at 3 days displayed the deficient phenotype, especially in the oocytes (Figure 4A). The oocytes did not have the typical banana shapes in their ovaries, while the ds*GFP*-treated females had normal ovaries containing banana-shaped oocytes (Figure 4B). Then, we calculated the egg number of dsRNA-treated females which mated with non-injected males. The result showed each ds*GFP*-treated female laid 100–150 eggs within ten days but that the ds*CPR*-treated females laid very few eggs in the rice seedlings (Figure 4C).

### 2.3. Effect of CPR Knockdown on CHC Levels

**Figure 5:**
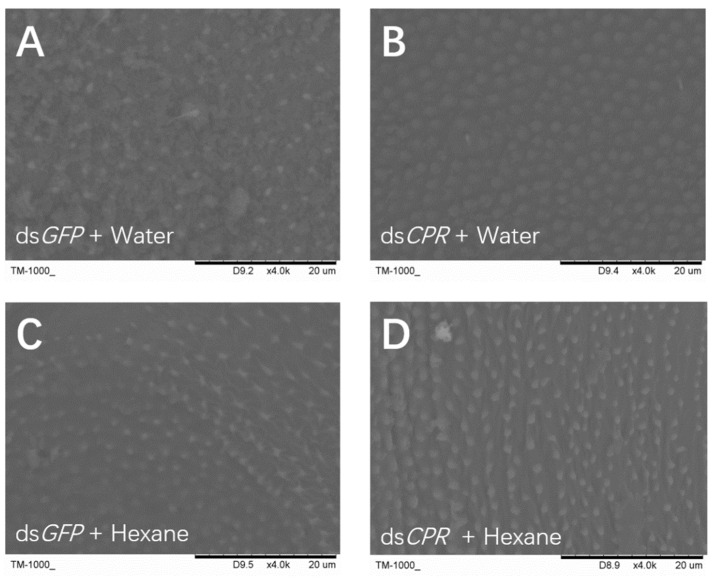
Scanning electron microscope analysis of the outermost layer in the abdominal surface. The 4th-instar nymphs at 48 h after molting were injected with dsRNAs and the 5th-instar nymphs at 48 h after emergence were collected for SEM. (**A**) ds*GFP*-injected insects were washed with water; (**B**) ds*CPR*-injected insects were washed with water; (**C**) ds*GFP*-injected insects were washed with hexane; (**D**) ds*CPR*-injected insects were washed with hexane. The 4th abdominal segment of the nymphs are shown.

To confirm the waterproofing and water retention functions of the *CPR* gene in the insect cuticle, we used scanning electron microscopy to observe the outermost layer of the abdominal cuticles of the ds*GFP*- and ds*CPR*-injected *N. lugens*. The images showed that a lipid layer covered the cuticle of ds*GFP*-treated *N. lugens* nymphal abdomen, suggesting its functions associated with the waterproofing and water retention (Figure 5A). However, the lipid layer was thin on the cuticle surface of the ds*CPR*-treated nymphal abdomen (Figure 5B). In the controls, the lipid layer was disrupted by n-hexane treatment in both ds*GFP*- and ds*CPR*-injected nymphs (Figure 5 C, D).

**Figure 6:**
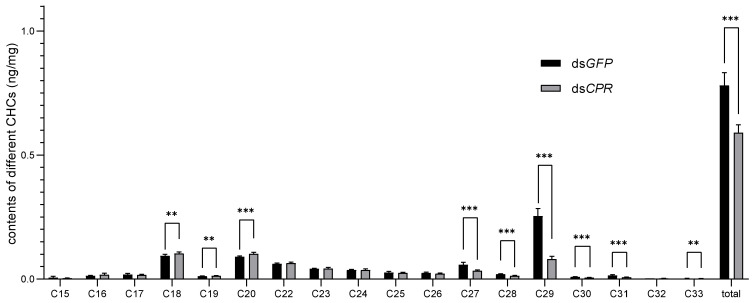
Determination of CHC contents in *N. lugens* female adults following dsRNA injection. The contents of total hydrocarbons and straight-chain alkanes with different lengths (C15–C33) in female adults were analyzed by GC–MS. The results were calculated from five biological replicates (nanograms per milligram of fresh body mass ± SE). The asterisks ** and *** indicate significant differences at *p* < 0.01 and *p* < 0.001 between ds*CPR* and ds*GFP*-treatments (Student’s *t*-test).

To further confirm whether the *CPR* gene knockdown caused a change in the CHC and a thinning in the lipid layer, we investigated the CHC levels in the ds*GFP*- and ds*CPR*-injected females 48 h after eclosion using gas chromatography–mass spectrometry (GC-MS). The results showed that the CHCs in adult females consisted of n-alkanes with various chain lengths, from C15 to C33, with C21 (n-heneicosane) used as an internal standard. The main component of CHCs in ds*GFP*-injected females was C_29_H_60_, which accounted for 32.55% of the total CHCs (Table 1). The ds*CPR* injection significantly decreased the total level of CHCs by 24.36%. The levels of C18, C19, and C20 were significantly increased by 11.11%, 13.04%, and 12.55%, and the levels of C27, C28, C29, C30, C31, and C33 were significantly decreased by 8.17%, 41.77%, 30.93%, 68.31%, 31.78%, 51.07%, and 21.20%, respectively (Figure 6). The proportion change of each CHC content was distinct. The proportion of n-alkanes with a chain length ≤ C26 increased by more than 20% (21.40–89.24%). However, the proportion of n-alkanes with a chain length of C27–C31 decreased. Among them, the proportion of C29 (as the main component) decreased by 58.1% (Table 1). The CHC profile changed dramatically, with the proportion of C15–C26 n-alkanes increasing from 53.94% to 75.48% and the proportion of C27–C33 n-alkanes decreasing from 46.06% to 24.52%.

## 3. Discussion

As an electron donor of P450s, the *CPR* gene plays a critical role in the metabolism of endogenous and xenobiotic substrates [5]. A previous study predicted the structure of the deduced CPR protein in *N. lugens*, which contains four conserved structural features: the N-terminal transmembrane region and the FMN-, FAD-, and NADP-binding domains [9]. *N. lugens CPR* shares a high sequence identity with the *Drosophila melanogaster* homolog. It is known that *D. melanogaster CPR* is involved in CHC biosynthesis and embryo development [6,7]. We suppose that *N. lugens CPR* may have potential functions in CHC biosynthesis and embryo development. In this study, we characterized the physiological function of the *CPR* gene in *N. lugens* by RNAi. The knockdown of the *CPR* transcript levels changed the CHC profile in female adults, leading to the function loss of waterproofing and water retention and inhibiting the embryo development in the female ovaries.

The *CPR* transcripts throughout all the developmental stages showed very high levels in the eggs, the 1st-instar nymphs, and the adults, suggesting that *CPR* might be involved in embryo development. The *CPR* transcripts in different tissues were at much higher levels in the ovaries and oocytes than in the other tissues, further indicating that *CPR* played an important role in oocyte maturity and development in adult ovaries.

To understand the functions of the *CPR* gene, we performed RNAi to knock down the transcript levels of *CPR*. The results showed that *CPR* knockdown led to waterproofing and water retention loss and generated lethal phenotypes, including molting failure and abdominal shrinkage. The body weights of *CPR*-knocked-down *N. lugens* were lighter than those of the ds*GFP*-treated controls, suggesting that the water retention function of the insect cuticle was lost. To better understand the physiological functions, we observed the survival of the dsRNA-injected *N. lugens* under different relative humidities. The survival time of the ds*CPR*-injected insects was much shorter than that of the ds*GFP*-injected ones. Under desiccation condition, the survival rates of ds*CPR*-injected insects dropped rapidly, and the median survival time was only 40% that of the control group. With the relative humidity increased to 50 ± 5%, the median survival time of dsRNA-treated *N. lugens* rose, but the median survival time of the ds*CPR*-injected ones was still much shorter than that of the control. Under a high relative humidity, no death was found in the ds*GFP*-injected *N. lugens* within 16 h, indicating that the water retention function was efficient. These results confirmed that *CPR* is involved in waterproofing and water retention functions. Previous studies have demonstrated that the CYP4G family is involved in the cuticle hydrocarbon biosynthesis [7,26]. As an indispensable component of the P450/CPR system, CPR may infect the CHC profile in a similar way and lead to the loss of waterproofing and water retention functions.

In the CHC profile assay, we first observed the lipid layer in the outermost cuticle of dsRNA-treated *N. lugens* insects. The lipid layer in the cuticle is the shield protecting the insects under humid or desiccated environments [23]. In this study, we found that the knockdown of *CPR* impaired the lipid layer and resulted in water loss under desiccation conditions and water being retained in the cuticle under humid conditions. Subsequently, we compared the CHC profiles of dsRNA-treated *N. lugens* female adults. *CPR* knockdown significantly increased the C18, C19, and C20 contents; increased the C16–C26 contents to a certain extent; and significantly decreased the C27, C28, C29, C30, C31, C33, and total CHC contents. The proportion of CHCs with a carbon chain length ≤ C26 increased by nearly 50%, and the proportion of C27–C33 n-alkanes decreased by nearly 50%. Our data indicated that the decreased total CHCs (especially C29, as the main component) and the change in the CHC profile caused the lipid layer to become thin and led to the failure of waterproofing and water retention. These results imply that the CHCs with a carbon chain length ≥ C27 may be the main functional aspects of waterproofing and water retention. The change in the CHC contents is likely to be a reason for the function loss of waterproofing and water retention.

In addition to the waterproofing and water retention functions, in this study we found for the first time that *CPR* knockdown generated deficient ovaries with deformed oocytes. These observations indicate that the *CPR* gene may participate in the regulation of embryo development and oviposition. We will clarify the function of *CPR* in female ovaries in future experiments to reveal the functional link between *CPR* and oocyte maturity and development in female ovaries. 

## 4. Materials and Methods

### 4.1. Insects

*N. lugens* populations were obtained and maintained in our lab, as previously described [27]. The *N. lugens* populations were originally collected from a rice field in the Huajiachi Campus of Zhejiang University, Hangzhou, China. The insects were maintained for at least 50 generations at 26 ± 0.5 °C with a 50 ± 5% relative humidity on rice seedlings (*Oryza sativa* strain Xiushui 134) under a 16:8 h light/dark photoperiod.

### 4.2. Cloning of a N. lugens CPR cDNA

The *CPR* cDNA sequence was obtained by searching the *N. lugens* transcriptome database in the Sequence Read Archive database (http://www.ncbi.nlm.nih.gov/sra, accessed date: 7 January 2019) (SRA accession number SRX023419) [28]. Total RNAs were extracted from the whole bodies of the various developmental stages using RNAiso Plus Total RNA extraction reagent (Takara, Dalian, China). First-strand cDNA was synthesized using HiScript^®^ II Q RT SuperMix for qPCR with gDNA wiper Mix (Vazyme Biotech, Nanjing, China). A pair of primers (sense: 5′-ATGGAGGTGGAGGCTGACTTAG-3′; antisense: 5′-TCAACTCCATACGTCGGCCG-3′) were designed to amplify the complete open reading frame (ORF) of the *CPR* gene. PCR was performed using 2 × Phanta^®^ Max Master Mix (Vazyme Biotech, Nanjing, China). The PCR product was cloned into the pMD19-T vector (Takara, Dalian, China) and sequenced by Sanger sequencing (Tsingke Biotech, Beijing, China).

### 4.3. Quantitative Real-Time qPCR (qRT-PCR) Analysis

Developmental stage- and tissue-expression patterns of the *CPR* gene were examined by qRT-PCR, as previously described [27]. Briefly, total RNAs were extracted from different tissues of *N. lugens* adults (fat body, gut, integument, testis, ovary, and oocyte) and the different developmental stages (eggs, nymphs, and adult males and females). The RNAs were reverse transcribed using a HiScript^®^ II Q RT SuperMix for qPCR with gDNA wiper Mix (Vazyme Biotech, Nanjing, China). qRT-PCR was performed in a 20 μL reaction volume containing 2 μL of 10-fold diluted cDNA, 0.6 μL of each primer and 10 μL of ChamQ SYBR Color qPCR Master Mix (Vazyme Biotech, Nanjing, China) using a Bio-Rad CFX96 real-time PCR system (Bio-Rad, Hercules, CA, USA). The gene-specific primers (sense: 5′-CTCGTGCCAGTCTTCATC-3′; antisense: 5′-CATACTCCTCAAGTTCCTCTT-3′) were used and the *N. lugens* 18S ribosomal RNA gene (GenBank No. JN662398) was used as an internal control to normalize the target gene expression using the following primers: 5′-CGCTACTACCGATTGAA-3′ (sense) and 5′-GGAAACCTTGTTACGACTT-3′ (antisense). The qRT-PCR reaction was performed using the following parameters: 95 °C for 30 s followed by 40 cycles at 95 °C for 5 s and 60 °C for 30 s. Three independent biological replicates were used, and each biological replicate included three technical replicates. The relative transcript levels were evaluated using the 2^−ΔΔCt^ method [29].

### 4.4. RNAi

Double-stranded RNA (dsRNA) of the *CPR* gene and an *Aequorea victoria* green fluorescent protein (GFP) gene used for control were individually synthesized by in vitro transcription with PCR-generated DNA templates using a MEGAscript T7 Transcription Kit (Vazyme, Nanjing, China). The primers used for dsRNA synthesis were sense-1: 5′-taatacgactcactatagggagaATGCCAAGAACGATGATGA-3′ with antisense-1: 5′-taatacgactcactatagggagaGAATAGTAACGACACTGAAGG-3′ and sense-2: 5′-taatacgactcactatagggagaCCGACAAGAAGCCATCTC-3′ with antisense-2: 5′-taatacgactcactatagggagaGCCGATAACATTCCATAGTTC-3′. The quality and concentration of dsRNAs were determined using agarose gel electrophoresis and a Nanodrop 2000 spectrophotometer (Thermo Scientific, Wilmington, DE, USA). The microinjection of dsRNAs was carried out as previously described [25,30,31]. The 4th- and 5th-instar nymphs were used for RNAi. Both the 4th- and 5th-instar nymphs were injected with ~10 or 20 nL of the dsRNA (5 ng/μL). The treated insects were kept on fresh rice seedlings at 26 ± 0.5 °C with a 50 ± 5% humidity under a 16:8 h light/dark photoperiod. Ten insects were randomly collected to evaluate the RNAi efficiency 3 days after dsRNA injection using qRT-PCR. Other insects were used for the calculation of survival rates and for the observation of development.

### 4.5. Measurement of Body Weight in dsRNA-Injected Insects

The 5th-instar nymphs were injected with ds*CPR* or ds*GFP*. Ten adult females, 48 h after eclosion, were pooled and used as independent samples for the measurement of body weight. Nine biological replicates were used for each dsRNA treatment. The ds*GFP*-injected adults were used as controls. Statistical analyses of the body weights between the different groups were tested using the Student’s *t*-test. *p* < 0.01 was considered to be statistically significant.

### 4.6. Oviposition Experiment

Oviposition was observed according to Peng et al. [32]. Briefly, the 5th-instar nymphs were microinjected with ds*CPR*. ds*GFP*-injected nymphs were used as a control. The insects were reared on fresh rice seedlings until emergence. Mating was performed between a single newly emerged female that had been injected with ds*CPR* and a single non-injected male adult. As a control, mating was performed between a single newly emerged female that had been injected with ds*GFP* and a single non-injected male adult. The mating was performed in a long glass tube containing three leaf-stage fresh rice seedlings (6.5 ± 0.5 cm long) at 26 ± 0.5 °C with a 50% ± 5% relative humidity under a 16:8 h light/dark photoperiod for 5 days. Then, the female and male adults were moved to a new glass tube containing three leaf-stage fresh rice seedlings for laying eggs for 5 days. The eggs in the rice leaf sheaths were dissected and counted. Biological replicates were used for each mating (n = 8 ♀ × ♂). The statistical significance of the number of laid eggs in the different treated groups was tested by a Student’s *t*-test. *p* < 0.01 was considered statistically significant. 

### 4.7. Waterproofing and Water Retention Assay

The 4th-instar nymphs were injected with ds*CPR*. ds*GFP*-injected nymphs were used as a control. The newly molted 5th instar nymphs were collected and kept on fresh rice seedlings in a plastic container at 26 °C ± 0.5, with a 50% ± 5 relative humidity, under a 16:8 h light/dark photoperiod, as described previously [20]. Water was sprayed onto the rice seedlings using a mini-sprayer. Insects were pressed into water contained in glass tubes. For a relative humidity treatment assay, the 5th-instar nymphs were injected with ds*CPR* and ds*GFP*. Nymphs were subjected to deferent relative humidity treatment using <5%, 50% ± 5, and >95% relative humidity at 26 °C ± 0.5, without food. The survival rates of the treated nymphs were calculated every 30 min.

### 4.8. Scanning Electron Microscope (SEM) Analysis

For the SEM analysis, the ds*CPR*- or ds*GFP*-injected 5th-instar nymphs were immersed in 200 μL of n-hexane or water for 1 min 48 h after molting. Then, insects were placed on a stub and dried in an ion sputter (Hitachi, Tokyo, Japan) under a vacuum [21,30]. After gold sputtering, the outermost layer of the insects was directly observed under SEM (TM-1000, Hitachi, Tokyo, Japan), as previously described. Three insects were observed for each treatment. 

### 4.9. Extraction and Quantification of CHCs

The 5th-instar nymphs were injected with ds*CPR*. ds*GFP*-injected nymphs were used as a control. CHCs were extracted from the females at 48 h after eclosion following a procedure used in our previous study [20,21,33]. Briefly, 10 females were immersed in 200 μL of n-hexane with 500 ng of n-heneicosane (C21) as an internal standard. The solvent was stirred gently for 3 min and then drawn with a glass Pasteur pipette into a clean chromatography vial. We repeated this procedure twice, then finally used 200 μL of hexane to rinse the nymphs and vial. We combined all the extracted hexane and dried it to a volume of 200 μL under high-purity nitrogen gas. Then, the hexane extracts were loaded onto an ∼300 mg silica gel (70e230 mesh, Sigma-Aldrich, Louis, MO, USA) mini-column in a glass wool-stoppered Pasteur pipette. The HC fraction was eluted with 2 mL of hexane, left to dry absolutely under nitrogen gas, and resuspended in 100 μL of hexane. The samples were analyzed on a TRACE 1310 (Thermo Scientific, Waltham, MA, USA) gas chromatograph (GC) equipped with an ISQ single quadruple MS and interfaced with the Xcalibur 2.2 software. The constant flow of helium was 1 mL/min. A splitless injection of 1.0 μL was carried out in a 30 m × 0.32 mm × 0.25 μm HP-5MS UI capillary column (Agilent Technologies, Santa Clara, CA, USA). The temperature program was operated at 60 °C for 2 min, then at 5 °C/min to 320 °C, then held for 10 min. The temperatures of the injector and detector were set at 300 and 280 °C, respectively. Mass detection was run under EI mode with a 70 eV ionization potential and an effective m/z range of 45–650 at a scan rate of 5 scan/s.

## 5. Conclusions

In this study, we confirmed that *CPR* is a multifunctional enzyme that participates in various biological processes. We verified that *CPR* is essential for waterproofing and water retention in *N. lugens*. Our novel findings reveal that *CPR* is required for embryo development. These findings expand our knowledge of the role of *CPR* in CHC biosynthesis and embryo development. This work establishes a functional link between CHC biosynthesis and embryo development, which may be a potential molecular target for the biological control of rice pests in the future. 

## Figures and Tables

**Table 1:** CHC profile of N. lugens female adults.

CHC	ds*GFP*	ds*CPR*	Content Change Rate	ds*GFP* Percentage of the Total	ds*CPR* Percentage of the Total	Proportion Change Rate
C15	0.005789 ± 0.005148	0.003344 ± 0.001431	−42.25%	0.74%	0.57%	−23.65%
C16	0.01252 ± 0.002376	0.017922 ± 0.006255	43.14%	1.60%	3.04%	89.24%
C17	0.018235 ± 0.004875	0.016934 ± 0.002075	−7.14%	2.34%	2.87%	22.77%
C18	0.093074 ± 0.006788	0.103419 ± 0.006303	11.11%	11.93%	17.52%	46.90%
C19	0.011544 ± 0.001355	0.013049 ± 0.000938	13.04%	1.48%	2.21%	49.44%
C20	0.089822 ± 0.003363	0.101096 ± 0.00619	12.55%	11.51%	17.13%	48.80%
C22	0.06181 ± 0.002839	0.064111 ± 0.004132	3.72%	7.92%	10.86%	37.13%
C23	0.041042 ± 0.00212	0.042432 ± 0.004224	3.39%	5.26%	7.19%	36.68%
C24	0.036321 ± 0.002465	0.036459 ± 0.004968	0.38%	4.65%	6.18%	32.71%
C25	0.026527 ± 0.004492	0.024414 ± 0.002927	−7.96%	3.40%	4.14%	21.68%
C26	0.02426 ± 0.004057	0.022278 ± 0.002021	−8.17%	3.11%	3.77%	21.40%
C27	0.057761 ± 0.010241	0.033636 ± 0.003238	−41.77%	7.40%	5.70%	−23.01%
C28	0.019489 ± 0.002171	0.013462 ± 0.001666	−30.93%	2.50%	2.28%	−8.68%
C29	0.254038 ± 0.030532	0.080512 ± 0.011656	−68.31%	32.55%	13.64%	−58.10%
C30	0.008287 ± 0.001302	0.005653 ± 0.000758	−31.78%	1.06%	0.96%	−9.81%
C31	0.014764 ± 0.002829	0.007224 ± 0.000978	−51.07%	1.89%	1.22%	−35.31%
C32	0.002795 ± 0.000268	0.002523 ± 0.0004	−9.73%	0.36%	0.43%	19.34%
C33	0.002263 ± 0.000237	0.001783 ± 0.000497	−21.20%	0.29%	0.30%	4.17%
Total	0.780341 ± 0.052279	0.590251 ± 0.032017	−24.36%	100.00%	100.00%	0.00%

Adult females at 48 h after emergence were collected for the analysis of the CHC profiles. The results were calculated from five biological replicates (nanograms per milligram of fresh body mass ± SE).

## Data Availability

Not applicable.

## Supplementary Materials

The following supporting information can be downloaded at https://www.mdpi.com/article/10.3390/ijms23052875/s1: Video S1. Phenotype of RNAi. ds*CPR* was injected in the 4th-instar nymphs at 48 h after hatching. Video S2. Phenotype of RNAi. ds*GFP* was injected in the 4th-instar nymphs at 48 h after hatching.
